# Profiles of executive functions in middle childhood and prediction of later self-regulation

**DOI:** 10.3389/fpsyg.2024.1379126

**Published:** 2024-05-09

**Authors:** Ariadne Brandt, Rebecca Bondü, Birgit Elsner

**Affiliations:** ^1^Department for Developmental Psychology, University of Potsdam, Potsdam, Germany; ^2^Department for Developmental, Pedagogic and Family Psychology, Psychologische Hochschule Berlin, Berlin, Germany

**Keywords:** executive functions, self-regulation, middle childhood, person-oriented approach, latent profile analysis, cognitive development

## Abstract

Most research on the development of executive functions (EF) has applied variable-oriented approaches, neglecting the potential inter- and intraindividual interplay of these capacities. In a person-oriented approach, the present study identified varying profiles of performance for three cool EF facets: inhibition, working-memory updating, and cognitive flexibility, as well as two hot EF facets: affective decision-making and delay of gratification, in a community sample of 1,657 children (T1; age: 6–11 years, *M* = 8.36 years, 52.1% female) via latent profile analysis. The best-fitting model allowed for partial dependence across the three cool EF and included four profiles: *all-average* (69.4% of the children), *low-delay* (19.0%), *regulated-decision-making* (7.0%), and *low-inhibition* (4.6%). Age, binary sex, socio-economic status, multilingualism, and processing speed were identified as significant characteristics of EF profile membership. Longitudinally, a higher probability of belonging to the *low-inhibition* profile predicted lower rates of the self-regulatory outcome of inhibitory control 1 year later, while belonging to the *regulated-decision-making* profile predicted lower rates of the ability to plan and organize 3 years later. These results not only demonstrate the existence of subgroups with different concurrent within-person expressions of EF performance, but also identify related characteristics and longitudinal outcomes of subgroup membership. In turn, these conclusions stress the importance of person-oriented research to inform on differing weaknesses or strengths in EF performance for varying individuals, thereby providing valuable insight for educational and clinical research into the design of effective personalized support or interventions during middle childhood.

## Introduction

Enrolling in elementary school typically marks an important transition in childhood, with self-regulation through executive functioning being a key component to success for the acquisition of new knowledge and skills in the classroom as well as new social relationships and competencies (Harms et al., 2014; Robson et al., 2020; Fernández García et al., 2021). Executive functions (EF) are a set of cognitive facets that enable monitored and controlled actions (Zelazo and Cunningham, 2007; Welsh and Peterson, 2014; Friedman and Miyake, 2017). Tapping into these functions, in turn, facilitates complex self-regulation (SR), including emotional self-control or long-term planning (Robson et al., 2020). Although the development of EF facets has been discussed rigorously (for a review, see Karr et al., 2018), little is known about their interpersonal variation (i.e., between-subject differences across EF expression) and concurrent intrapersonal variation (i.e., between-facet differences within one individual) during middle childhood. The prevailing variable-oriented analyses mostly fail to consider the existence of subgroups of individuals who differ in their performance across a comprehensive set of EF facets, and how these unfold across childhood development. This is due to the nature of variable-oriented statistics such as factor- or regression analyses, which necessitate the aggregation of data across an entire population for the analysis of generalized relationships between the variables of interest. This can lead to misrepresentations—particularly for smaller subgroups within the overall population that might deviate from a given norm (von Eye and Bergman, 2003). To avoid this issue, the disaggregation of data via person-oriented analyses and methods including cluster-and latent profile analyses allows for more accurate inferences about subgroups of individuals. The present person-oriented study therefore aimed to identify specific latent profiles of five EF facets within a community sample of 6- to 11-year-old children, the resulting profiles’ varying characteristics, and the predictive value of EF profile membership for later SR abilities across 3 years.

The EF facets that emerge throughout childhood include *inhibition*, the ability to control dominant or automatic reactions in favor of goal-oriented, adapted responses (Miyake et al., 2000; Friedman and Miyake, 2017); *working-memory updating*, the ability to revise and recode information and representations that are relevant for a current task (Miyake et al., 2000); and *cognitive flexibility*, the ability to switch quickly between relevant rules or mental sets according to tasks or expectations (Miyake et al., 2000; Röthlisberger et al., 2010). These EFs are considered “cool” because they manifest in rational, decontextualized, and non-emotional tasks (Peterson and Welsh, 2014). In contrast, “hot” EFs are more complex, contextualized, and emotionally-driven (Hongwanishkul et al., 2005; Peterson and Welsh, 2014; Poon, 2017; Lensing and Elsner, 2018). Hot EFs include *affective decision-making*, the tendency to be influenced by possible rewards and emotional biases when faced with a choice, even if these actions entail disproportionate risks (Crone and van der Molen, 2004; Cauffman et al., 2010); and *delay of gratification*, the ability to decide against short-term reward in favor of long-term benefit (Wulfert et al., 2002; Peterson and Welsh, 2014). Challenges for research on EF development arise from the overlap and interplay of these five facets in daily life (Hongwanishkul et al., 2005; Peterson and Welsh, 2014; Wang et al., 2020).

In accordance with the prevailing age differentiation hypothesis, EF skills become increasingly distinct and sophisticated across childhood development (Lee et al., 2013; Brydges et al., 2014). Parallel to their differentiation, EF facets become more dissociable from one another when using experimental paradigms in middle and late childhood (Lehto et al., 2003; Prencipe et al., 2011; Brydges et al., 2014; Groppe and Elsner, 2014). Inhibition (and sometimes working-memory updating) are frequently mentioned as the cool EF to mature the fastest in this phase (Lehto et al., 2003; Best and Miller, 2010). Fittingly, inhibition showed the smallest change rates in a large cohort of 11- to 19-year-olds, implicating a more protracted specification of working-memory updating and cognitive flexibility in comparison (Boelema et al., 2014). A cross-sectional study with 7- to 15-year-olds also found significant age effects for all three cool EFs (Xu et al., 2013). On closer scrutiny, there were strong correlations among the three cool EF facets, promoting a one-factor cool EF model and thereby indicating little differentiation for both the 7- to 9-year-olds and the 10- to 12-year-olds in the study. However, for the oldest participants (13–15  years) the cool EFs inhibition, working-memory updating, and cognitive flexibility became distinguishable, and a three-factor model provided an improved fit. While the development of cool EF seems to follow a linear pattern (Best and Miller, 2010), some findings suggest a more complicated trajectory for hot EF characterized by protracted development and by particular caveats (i.e., a performance dip) during adolescence, with adult-like task performance achieved around 17–18  years (Hooper et al., 2004; Zelazo and Carlson, 2012; Tsermentseli and Poland, 2016; Cortes-Patino et al., 2017). A developmental cascade would suggest that the early improvement of more basal cool EF like inhibition may initiate the specification of increasingly hot EF and complex SR abilities including anger regulation and long-term planning abilities later on in middle childhood and adolescence (Best and Miller, 2010; McAuley and White, 2011; Prencipe et al., 2011). To date, little is known about the exact order of differentiation of hot EF facets and their potential intercorrelation with individual cool EF abilities.

According to the unity/diversity framework, the three cool EF facets are moderately correlated in adulthood, while remaining independent skills within a 3-factor structure that emerges throughout childhood development (Miyake et al., 2000; Poon, 2017; Karr et al., 2018). The framework assumes that cool EF share underlying functional commonalities (McAuley and White, 2011; Hartung et al., 2020) and related neural networks within the prefrontal cortex (Li et al., 2015; Wang et al., 2020). The framework has since been extended to include hot EFs (Peterson and Welsh, 2014; Tsermentseli and Poland, 2016), but findings regarding a hot EF factor structure are contradictory: decision-making and delay of gratification do not always associate and are sometimes found to be inversely correlated during early childhood and up until youth (Hongwanishkul et al., 2005; Lee et al., 2013; Groppe and Elsner, 2014; Li et al., 2015; Poon, 2017; O’Toole et al., 2018). Reasons for these inconsistencies include the protracted development of hot EF and methodological discrepancies such as differing EF-combinations or experimental tasks used across studies (Peterson and Welsh, 2014; Friedman and Miyake, 2017; Poon, 2017; Eisenberg et al., 2019). Lastly, by not taking inter- and intraindividual differences into account, these variable-oriented investigations risk the loss of vital information. These open issues stress the importance of person-oriented research to better understand how cool and hot EF facets specialize and interact throughout childhood across and within different individuals.

Latent person-oriented research is becoming more popular within developmental and cognitive psychology as it identifies meaningful subgroups within larger samples that deviate in their performance or characteristics from the sample’s mean and would otherwise be overlooked. Within a large cohort of children between 9 to 10 years, Chaku et al. (2022) pinpointed four subgroups or latent profiles with different expressions of cool EF facets. Along with an average, a high, and a low EF profile, the authors identified a low inhibition profile. These EF profiles were characterized by gender and socio-economic status (SES), and predicted later differences in reported social, attentional, and externalizing problems. Another study restricted to multilingual preschoolers identified three profiles consisting of the three cool EF facets: these profiles were mainly characterized by the manifestation of cognitive flexibility (above- or below-average performance), likely due to the unique sample’s linguistic abilities which were argued to influence the cool ability to shift flexibly between rules and schemas (Relyea et al., 2023). While other developmental studies also utilized EF in the generation of unique cognitive profiles, they focused on specific populations (i.e., children with ADHD or ASD; Dajani et al., 2016) or neglected certain EF facets, which leads to a poor differentiation of EF performance within the respective profiles (Litkowski et al., 2020). While initial attempts to profile EF in childhood have been made, the present study makes an important contribution by investigating a comprehensive set of both cool and hot EF skills to provide a detailed picture of meaningful subgroups of children who differ in their executive abilities both intra- and interindividually.

Most studies on EF development have addressed how latent factors differentiate with increasing age while correlating with demographics and outcomes. In doing so, research remains variable-oriented and comments on the structural change of EF while assuming that the sample at hand belongs to a single population (Karr et al., 2018). This assumption is contrasted by findings showing that demographic variables including age, binary sex, SES, and multilingualism are associated to childhood EF performance. Older age and higher SES are associated with increased EF abilities in middle childhood in general (Lensing and Elsner, 2018; Wang et al., 2020; Holl et al., 2021; Williams and Bentley, 2021). In particular, multilingualism is associated with increased cognitive flexibility, due to multilingual children’s early need to switch between the languages they learned dependent on the context they are in (Relyea et al. 2023). Sex is differentially associated with EF performance, with girls typically excelling at cool EF in childhood and boys excelling at hot EF in adolescence (Peterson and Welsh, 2014; Berthelsen et al., 2017). Finally, processing speed is considered a stable and strong correlate of intelligence (Luciano et al., 2001; Boelema et al., 2014) and is central for most tasks that require executive control or self-regulation (Holl et al., 2021). These differences further stress the need for person-oriented investigations into EF structure, dependent on respective subgroup differences in performance and associated characteristics.

Despite a structural overlap between developing EF facets observed in variable-oriented research (Zelazo and Cunningham, 2007), a dissociation seems to exist regarding their respective life outcomes. Cool EF are positively associated with intelligence as well as academic or vocational success (Röthlisberger et al., 2010; Diamond, 2014; Peterson and Welsh, 2014; Poon, 2017; Jacobson et al., 2018). Hot EF are positively associated with social competence and negatively with risk-taking behavior and health outcomes including obesity and addiction (Wulfert et al., 2002; Peterson and Welsh, 2014; Lensing and Elsner, 2017; Orm et al., 2022). To date, few studies have investigated the predictive value of basal EF facets on the emergence of more complex SR abilities. Within a study-based theoretical model, Diamond (2014) proposed that the three cool EF predicted the higher-order ability to plan, which was in turn linked to reasoning and problem-solving abilities. Other authors consider planning to be a fourth cool EF, which can successfully be integrated within factorial models together with the original three cool EF during development (7- to 18-year-olds; Laureys et al., 2022). Either way, an association between cool EF and the SR abilities of planning and organizing seems inherent. In a longitudinal study, Shoda et al. (1990) found that a behavioral measure of the hot EF delay of gratification at 4 years predicted later self- and parent-reported SR competencies including the ability to plan but also the control of emotions in frustrating situations at 15 and 18  years. Fittingly, Zelazo and Cunningham (2007) stressed the direct connection between emotionally motivated EF centered around reward or punishment, and emotional control and reactivity *per se*. These findings were disputed more recently in a replication study, which did not find that preschool delay of gratification related longitudinally to adolescent emotion regulation, but only to increased social skills and reduced problem behavior (Michaelson and Munakata, 2020). These short- and long-term effects remain difficult to disentangle because of the interdependence between facets of both basal EF and complex SR.

To summarize, studies have frequently used cross-sectional data (McAuley and White, 2011; Xu et al., 2013; Robson et al., 2020) or focused solely on cool EF (Lee et al., 2013; Hartung et al., 2020; Litkowski et al., 2020; Chaku et al., 2022) when investigating EF change. This has led to an imbalance in knowledge about development of hot EF (Peterson and Welsh, 2014). Most importantly, many studies have neglected the interpersonal variance within childhood development that may lead to different patterns and trajectories of performance *within* any given sample. The few studies which have utilized person-oriented approaches focused solely on cool EF and/or on specific subpopulations including multilingual children or children with ADHD (Dajani et al., 2016; Relyea et al., 2023). In other words, no study to date has investigated a comprehensive range of cool and hot EF in a person-oriented approach within a heteronormative community sample of school-aged children. Potential concurrent intra- and interindividual differences in EF are of particular interest during middle childhood because this age is marked by cognitive, behavioral but also by social and educational variance and change.

## The current study

**Figure 1:**
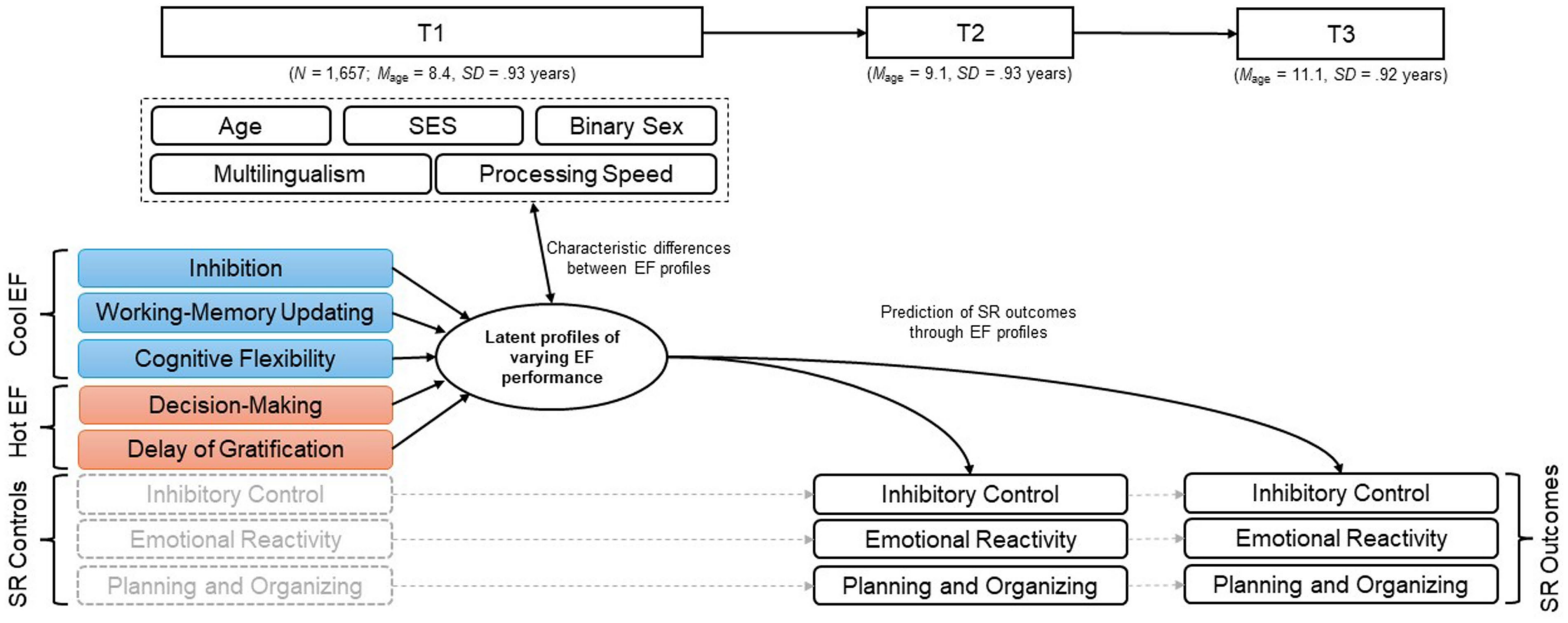
Overview of all time points (T1–T3) and all variables included in the current analyses. EF, executive functions; SR, self-regulation; SES, socio-economic status.

The present study aimed to identify person-oriented latent profiles of EF performance in a community sample of school-aged children (at T1; 6–11 years) and to examine the profiles’ predictive value across 3 years. Our objectives were first, to identify the prevalence and shape of different latent profiles of performance in the three cool EF facets inhibition, working-memory updating, and cognitive flexibility, and the two hot EF facets affective decision-making and delay of gratification. Second, we aimed to characterize the identified EF profiles at T1 by investigating how the children assigned to each profile differ across concurrent variables including age, binary sex, SES, multilingualism, and processing speed. Third, we sought to determine whether and how EF profile membership at T1 (6–11 years) would predict later parent- or teacher-reported performance on outcome measures of SR after about 1 year (T2) and 3 years (T3). These outcomes included inhibitory control, emotional reactivity, and planning/organizing ability.^1^ An overview of all variables and the planned analyses is found in Figure 1.

In line with our first aim, we predicted that school-aged children at T1 could be grouped into at least four latent profiles varying in their mean expression (i.e., higher or lower performance compared to the overall sample mean) of three cool and two hot EF facets (Chaku et al., 2022). According to the unity/diversity framework, we assumed that moderate intercorrelations between the behavioral measures of (cool vs. hot) EF would remain evident within the unique latent profiles’ shapes. Due to past latent models and observations that cool EFs, particularly inhibition and working-memory updating, mature prior to hot EFs (Lehto et al., 2003; Zelazo and Carlson, 2012; Karr et al., 2018; Wang et al., 2020; Chaku et al., 2022), the expected profiles included: (H1.1) an *all-low EF* profile and (H1.2) an *all-high EF* profile in which children consistently perform below or above the whole-sample average, respectively, across all five facets; (H1.3) a *high-cool, low-hot EF* profile in which children perform above-average on the three cool facets, but below-average on the two hot facets; and (H1.4) a *high-inhibition* or a *high inhibition and updating* profile in which children perform above-average on only these cool facets. These profiles would suggest different interrelations of individual developmental trajectories of cool and hot EF facets. The prevalence of the identified EF profiles in the community sample will be reported: we hypothesized (H2) that *high cool-low hot* and *high-inhibition* profiles may dominate, in line with the earlier development of the less complex cool EF before adolescence (Zelazo and Carlson, 2012).

Based on the above-described associations of EF development and demographic variables, we expected that identified latent profiles marked by above-average performance on EF facets would be characterized by (H3.1) higher ratios of children with increased age (Boelema et al., 2014; Wang et al., 2020), (H3.2) higher SES (Boelema et al., 2014; Lensing and Elsner, 2018), and (H3.3) higher information-processing speed (Boelema et al., 2014). We assumed higher ratios of (H3.4a) girls in profiles marked by above-average cool EF performance, and of (H3.4b) boys in profiles with above-average hot EF performance (Peterson and Welsh, 2014; Berthelsen et al., 2017; Chaku et al., 2022). Finally, we assumed (H3.5) higher ratios of children who speak more than one language in profiles marked by higher cool EF performance (Relyea et al., 2023). For profiles marked by below-average EF performance, we assumed the respective reverse expressions: more children with lower age, lower SES, and lower processing speed; more boys in low-cool and more girls in low-high EF profiles; and less multilingual children.

Regarding the predictive value of EF profile membership at T1 for parent- or teacher-reported outcome measures of SR at T2 and T3, we expected that (H4.1) belonging to a profile marked by above-average performance on cool EF facets should be positively related to later inhibitory control (Jacobson et al., 2018) and (H4.2) planning/organizing ability (Diamond, 2014; Friedman and Miyake, 2017). We additionally assumed that (H4.3) profiles with below-average hot EF facets should predict increased later emotional reactivity (Shoda et al., 1990; Castillo and Lopez, 2022). Alternative profiles at T1 including their concurrent characteristics and consequent outcomes will be explored, if found during modeling.

## Materials and methods

### Participants

Participants were recruited in schools for a mixed-methods and multi-informant longitudinal project focussed on intrapersonal developmental risks (PIER-Study*: Potsdamer Intrapersonale Entwicklungsrisiken Studie*). Children from 33 elementary schools throughout the Federal State of Brandenburg, Germany were recruited, tested, and followed up twice within about 3  years. At T1, *N* = 1,657 children (aged 6.23–11.33 years; mean age = 8.36 years, *SD* = 0.95, 52.1% female) in 1st to 4th grade took part. Furthermore, 1,424 class teachers and 1,316 parents completed surveys on their respective students/children. According to parental report, 6.8% of the sample came from a multilingual background and spoke an additional language other than German within their family home. SES was high, based on the parent-reported highest educational degree: for 45.5% of the sample, at least one parent had completed a higher-level education (i.e., university or college). For 19.3%, at least one parent had completed upper secondary schooling. For 34.5%, at least one parent reported completing some form of lower secondary school, and for 0.7% of the sample neither parent reported completing school. At T2 (ca. 9 months after T1; *M* = 273.3 days, *SD* = 55.5) we collected data from *N* = 1,608 children (mean age = 9.11 years, *SD* = 0.93, 51.8% female), *N* = 1,175 teachers, and *N* = 1,196 parents, and at T3 (ca. 32 months after T1; *M* = 989.9 days, *SD* = 76.8) from *N* = 1,531 children (mean age = 11.06 years, *SD* = 0.92, 51.4% female), *N* = 1,113 teachers, and *N* = 1,040 parents.

### Procedures

At all three measurement points, trained experimenters tested each child individually during the morning hours in an empty (class)room. Each child took part in two sessions lasting an hour each (mean days between sessions = 7.2, *SD*  = 7.6). The sessions contained a mix of experimental tests, questionnaires, and physiological measures. A total of four test blocks were randomized across two sessions in the order AB-CD or BA-DC across participants. Participants received small gifts (toys, stickers) and a voucher worth 7.50 Euros as compensation. Parents and class teachers completed questionnaires regarding the respective child’s academic achievement, personality and temperament, social skills, and self-regulatory abilities. Teachers received 5 Euros for their classroom funds per child for whom they provided information. Informed written consent was provided by both the children’s parents and the participating schools, and informed oral consent was obtained from each child. Procedures were approved by the research ethics board at the University of Potsdam as well as the Ministry of Education of Brandenburg, Germany. The current analysis was preregistered (OSF: https://doi.org/10.17605/OSF.IO/TEPXU).

### Measures

#### T1: EF measures

Each of the five EF facets was operationalized using one age-appropriate behavioral experimental task at T1. Brief descriptions are provided below, while more detailed accounts can be found in the study protocol (Warschburger et al., 2023).

**Inhibition (I)**. This cool EF facet was operationalized using the age-appropriate fruit-/vegetable-stroop task (Roebers et al., 2011), accounting for the limited reading capacity of elementary school children (Archibald and Kerns, 1999). Children were asked to name the colors of 25 items presented on a sheet as quickly as possible. The first sheet displayed 25 colored (blue, red, green, yellow) rectangles. The second sheet depicted 25 fruits and vegetables in their true colors (i.e., grapes—blue, strawberry—red, lettuce—green, banana—yellow). The third sheet depicted the same items in grey, and the fourth sheet in incongruent colors (i.e., banana—blue), and children were instructed to name the respective true color of the fruits and vegetables (i.e., banana—“yellow”) while ignoring the printed incongruent color. An interference score was generated: *time SHEET 4 − [(time SHEET 1 × time SHEET 3)/(time SHEET 1 + time SHEET 3)]* (Röthlisberger et al., 2010). Scoring high indicated a decreased ability to inhibit the prepotent and automated response of naming the items’ actual color on the fourth, incongruent sheet. The respective *z*-standardized scores were then reversed for better interpretability, so higher values indicated better inhibition.

**Working-memory updating (U)**. This cool EF facet was operationalized using the digit span backwards task of the German Version of the Wechsler Intelligence Scale for Children (HAWIK-IV; Petermann and Petermann, 2008). Children were asked to verbally repeat, in backward order, a string of digits read out by the experimenter. After one practice block, each child completed up to eight test blocks containing two strings of the same length. The first two strings each consisted of two digits, and when a child correctly repeated at least one string of each length successfully, the experimenter added one digit to the next string in the next block. The eighth and final test block contained two nine-digit strings. If the child failed to repeat both digit sequences of a given length backwards, the test was terminated. Each participant received a sum score of correct backward repetitions across all test blocks out of the maximum of 16 presented strings. Higher scores indicated better updating abilities.

**Cognitive flexibility (F)**. This cool EF facet was operationalized using the computerized feeding fish task (Roebers and Kauer, 2009). Children were requested to feed two fish (one multi-colored, the other single-colored) presented on the screen by pressing the X-and M-keys for the left and right fish, respectively. Children were instructed to feed the two fish in an alternating pattern, requiring them to remember which fish they had fed last. The task consisted of 46 trials, of which the first 2 trials and 22 further non-switch trials required an alternating response pattern (i.e., left–right–left). The remaining 22 trials were switch trials, in which the location of the fish changed, requiring a repetition of the previous response (i.e., left–left) and thus a spontaneous change in the previous answer pattern in order to maintain the rule of feeding alternately. Interstimulus intervals ranged from 300 ms to 700 ms. Reaction time (RT) was measured, and data was cleaned of all trials with a RT <200 ms (anticipatory error) or >5,271 ms (+3 *SD* s above the sample mean; omission error). This applied to only 1.52% of the data, resulting in an average number of 45.3 valid trials (out of the original 46; *SD*  = 1.64, range 23–46 trials) per participant. Mean RT on correct trials (switch and non-switch, omitting two establishment trials) was used, with faster RTs indicating better flexibility. We excluded the data of *n* = 118 children (7.3%) who answered below chance level (less than 22 correct answers), which may be indicative of task misunderstanding.

**Affective decision-making (ADM)**. This hot EF facet was operationalized using the computerized hungry donkey task (Crone and van der Molen, 2004), a child-friendly version of the Iowa gambling task. Children were confronted with four identical doors presented on a PC monitor. Each door could be opened using the keyboard (keys S, D, K, L for doors A, B, C, D, respectively). The children were instructed to collect as many apples as possible, hidden behind the doors, for a hungry donkey. A total of 60 trials (i.e., 60 key presses) were played. Upon each keypress, a certain number of green apples (gain) and red apples (loss) were displayed in the opened door, along with the current net score of apples collected. Unknown to the children, the doors had differing underlying contingencies: the unfavorable doors A and B yielded higher gains (+4 apples per trial), but also larger losses (−8 to −12 apples in 50% of the trials), resulting in long-term loss. The favorable doors C and D yielded lower short-term rewards (+2 apples per trials) but also smaller losses (−1 to −3 apples in 50% of the trials), resulting in long-term gain. Children were promised a prize marble if they managed to collect at least 20 apples for the donkey. A net score was calculated by subtracting the number of unfavorable doors from the number of favorable doors [(C + D) − (A + B)] chosen, assuming that children who succeeded at regulating their decision-making strategies and who learned from the consequences of their actions would adapt their behavior and start choosing the favorable over the unfavorable doors (Crone and van der Molen, 2004). The first 10 trials were excluded from the net score as they were considered necessary to explore the contingencies. A high score indicated more favorable choices and thus regulated rather than affective decision-making.

**Delay of gratification (D)**. This hot EF facet was operationalized using a behavioral task testing each child’s ability to deter immediate reward in favor for a more attractive but delayed reward (adapted from Wulfert et al., 2002). In 4 trials, children were presented with a small gift (i.e., a piece of candy) and asked whether they would like to receive the gift immediately (coded as 1 point) or rather receive more of the same gift (i.e., two pieces of candy) a week later (coded as 2 points). Depending on their decision, children received the promised gift promptly or during the second testing session. Children were additionally asked whether they liked each item offered to them (coded as 2 points) or not (coded as 1 point). In total, 97.2% of the sample liked all, or at least three of the rewards. Then each trial was weighted by multiplying the coded decision by the coded preference so delaying a liked item received double the valence (4 points) compared to delaying a non-liked item (2 points). A total score across the 4 trials (min. = 4, max. = 16) was used to calculate a ratio with a value closer to 1 indicating more frequent delay, even for attractive rewards.

#### T1: characteristics of EF-profile members

Parents reported on their child’s birth date (age at time of data collection was calculated accordingly) and binary sex. As a proxy for SES, the highest maternal and/or paternal educational degree was identified in the parent questionnaire on a categorical scale, with 1 indicating no educational qualification and 6 indicating a completed university or college degree. Additionally, parents were asked what language they used at home when talking to their child. If at least one parent spoke a language apart from German, we considered the child to be multilingual. Lastly, we measured processing speed with the digit symbol coding test of the German Version of the Wechsler Intelligence Scale for Children (HAWIK-IV; Petermann and Petermann, 2008). In this pen and pencil task, children were provided with a legend in which numbers (1 through 9 for children 8 years or older) or shapes (for children between 6–7 years) were paired with a simple geometric symbol. Children received a matrix of 60 numbers or shapes and filled in the corresponding symbol below each item as quickly as possible. We scored the maximum number of correctly drawn symbols within 120 s. If a child completed all 60 symbols in less than 120 s, they received additional points (max +5).

#### T1–T2–T3: self-regulatory measures (outcomes; parent and teacher report)

*Inhibitory control* was operationalized using 6 items from the corresponding subscale of the Temperament in Middle Childhood Questionnaire (TMCQ, Simonds, 2006) filled out by parents. The subscale captures an individual’s ability to act appropriately in unfamiliar or regulated situations and includes items such as “can stop themselves from doing things too quickly.” Answers were given on a 5-point Likert scale ranging from 1 (*does not apply*) to 5 (*applies*), so a higher mean score across all 6 items indicated higher inhibitory control. Internal reliability was acceptable within the sample for all time points (0.67 ≤ *α* ≥ 0.71).

*Emotional reactivity* was operationalized using the subscale *emotional control* of the Behavioral Rating Inventory of Executive Function, completed by parents. The subscale describes the ability to modulate and control emotional reactions appropriately (Gioia et al., 2016). English items were back-translated to German by two bilingual research assistants. The subscale consists of 10 items such as “small events trigger big reactions” answered on a 5-point Likert scale ranging from 1 (*never*) to 5 (*always*). Items were re-coded so that a higher score indicated lower reactivity, and a mean was calculated across all 10 items. Internal reliability was high within the current data for all time points (0.91 ≤ *α* ≥ 0.92).

*Planning and organizing* was operationalized using the subscale of the Behavioral Rating Inventory of Executive Function completed by class teachers (Gioia et al., 2016). Planning and organizing describes the ability to work in a structured and organized manner towards future goals or deadlines. English items were back-translated to German by two bilingual research assistants. Out of 10 original items, 8 were chosen according to factor loadings, including items such as “underestimates time needed to finish tasks,” answered on a 5-point Likert scale ranging from 1 (*never*) to 5 (*always*). Items were re-coded, so that a higher mean score across all 8 items indicated increased planning abilities. Internal reliability was high within the sample for all time points (0.92 ≤ *α* ≥ 0.96).

### Analysis plan

All analyses were carried out using SPSS (IBM Statistics, Version 29) and M*plus* (Muthén & Muthén, Version 8.7). To address the primary aim and the first two hypotheses (H1.1–H1.4; H2), latent profile analyses (LPA) were calculated to identify meaningful subgroups differing in performance across inhibition (I), working-memory updating (U), cognitive flexibility (F), affective decision-making (ADM), and delay of gratification (D) at T1. For best comparability, *z-*standardized values were considered. A series of LPA-models were estimated using mixture modeling ranging from 1 to 6 profiles, respectively. Sets of invariant models, free variance models, free residual covariance models, and unrestricted models were explored to identify the best fit (Johnson, 2021). Starting values and the number of iterations were adjusted to decrease the likelihood of reaching local maxima and to increase the likelihood for global solutions (Spurk et al., 2020). To control for non-normally distributed variables, an MLR estimator (maximum likelihood estimation with robust standard errors) was chosen. Profiles were only considered if they included more than 30 cases (approximately 2% of the sample) to ensure interpretability (Ferguson et al., 2020; Spurk et al., 2020). The series of models was compared, and the most ideal solution determined according to several fit indices including the log-likelihood parameter, sample-adjusted Bayesian information criterion (BIC), and Akaike’s information criterion (AIC). For all fit indices, smaller relative values indicated better fit of a current model, compared to other models. The Lo–Mendell–Rubin (LMR) test and the bootstrap likelihood ratio test were considered to quantify whether adding an additional profile to the model significantly (*α* < 0.05) increased the variance explained. Entropy was taken into account, with a value above .80 considered desirable to guarantee model certainty and accurate profile assignment (Ferguson et al., 2020). Parsimony was favored. Due to the sample’s broad age range, latent profile models were also tested post-hoc for 6–7.99 (*n* = 624) and for 8–9.99-year-olds (*n* = 984) separately. After determining that a 4-profile solution with partial cool EF dependence provided the best fit for both age groups separately (Supplementary Table S2) and that the profiles did not change in their respective shapes per age group (Supplementary Figure S1), analyses for the entire sample were reported to ensure statistical power. Controlling (i.e., clustering data) for school class membership using a sandwich estimator during LPA modeling did not lead to significant changes in model fit and was therefore not considered further.

To address H3.1–H3.5, the differentiating value of demographics including age, binary sex, and SES, as well as multilingualism and processing speed for profile membership was analyzed via a multinomial logistic regression using an R3Step automated approach and Bose–Chaudhuri–Hocquenghem (BCH) weighting to prevent large changes in profile membership and to account for measurement error and model uncertainty.

To investigate whether different EF-profiles had predictive power for future SR skills (H4.1–H4.3), reported mean scores of inhibitory control, emotional reactivity, and planning and organizing for T2 or T3, respectively, were regressed onto individual LPA membership probabilities at T1. In effect, each individual child received a probability for each profile, indicating how likely a membership within each profile for said individual would be. Separate hierarchical linear regressions for the SR outcomes and all correlated profile membership probabilities were carried out. In order to identify potential change within the respective time span, SR outcomes were controlled for their status quo at T1 for T2 predictions, and at both T1 and T2 for T3 predictions in step 1 of each model. Profile membership probabilities were added to each model in step 2. Collinearity statistics (bivariate correlations <0.80; variance inflation factor <5; tolerance >0.10; condition number <30) were considered to rule out multicollinearity.

As previous publications have discussed and established latent EF factor models within the current sample (Groppe and Elsner, 2014), these analyses were not repeated here. It was acknowledged that a cool EF-factor has been identified at T1, confirming that performance in the three cool EF tasks (I, U, F) is slightly to moderately intercorrelated. The two tasks associated with hot EF (ADM, D) did not form a factor across the available data—making a more detailed and individual analysis ever the more interesting.

#### Missing data

Due to the study’s longitudinal and multi-informant design, missing data and dropout rates are a relevant issue. Attrition within the sample was comparably low for a longitudinal cohort design (drop-out of 8% from T1 to T3 compared to a mean attrition of 26.5% as reported in a metanalysis of 143 studies; Teague et al., 2018). Data points that were missing *within* a single test battery were rare and considered missing completely at random. Not all parents and teachers completed a survey at T1, and larger proportions dropped out across T2 (parents: 9.1%, teachers: 17.5%) and T3 (parents: 13.0%, teachers: 5.3%). This data cannot be assumed missing at random. To avoid listwise deletion of data with missing independent predictors (i.e., profile characteristics), full information maximum likelihood estimation via Monte Carlo simulation was applied.

## Results

### Descriptives

Means, standard deviations, and ranges across all variables of interest are reported for the sample in Table 1. Between-subject *t*-tests (including equivalents accounting for inequality of variance) were carried out to identify binary sex differences across the variables. Boys and girls did not differ significantly in age, SES, or multilingualism. However, girls performed higher than boys on processing speed, *t*(1,645) = 6.06, *p* < 0.001, *d* = 0.30. Girls also exhibited significantly less interference than boys on the T1 inhibition task, *t*(1539.91) = −3.31, *p* < 0.001, *d* = 0.16, and reacted more quickly on the flexibility task while answering correctly, *t*(1520) = −3.53, *p* < 0.001, *d* = 0.18. However, girls scored significantly lower than boys on the affective decision-making task by choosing more unfavorable doors, *t*(1443.68) = −5.46, *p* < 0.001, *d* = 0.27. Likewise, girls delayed weighted decisions less frequently than boys in the delay of gratification task *t*(1,621) = −2.17, *p* = 0.03, *d* = 0.11. There were no significant sex differences for updating, *p* > 0.5. Lastly, parents and teachers reported that girls compared to boys had significantly higher rates of inhibitory control [T1: *t*(1308.63) = 4.64, *p* < 0.001, *d* = 0.26; T2: *t*(1,188) = 4.32, *p* < 0.001, *d* = 0.25; T3: *t*(1,060) = 3.45, *p* < 0.001, *d* = 0.21] and planning and organizing ability [T1: *t*(1,417) = 8.27, *p* < 0.001, *d* = 0.44; T2: *t*(1138.03) = 8.97, *p* < 0.001, *d* = 0.53; T3: *t*(1,106) = 6.75, *p* < 0.001, *d* = 0.41], but not emotional reactivity, all *p*-values > 0.10.

**Table 1:** Descriptive statistics of all study variables, including binary sex differences calculated via between-subject *t*-tests.

T1: characteristics of EF-profiles	Range sample [theoretical]	Total|*M* (*SD*)	Boys|*M* (*SD*)	Girls|*M* (*SD*)
Age [years]	6.23–11.33 [n.a.]	8.36 (0.95)	8.39 (0.98)	8.33 (0.92)
Socio-economic status^a^	1–6 [1–6]	5.05 (1.00)	5.06 (1.01)	5.04 (1.00)
Multilingualism	% of sample [n.a.]	6.8	6.7	6.8
Processing speed [score—digit symbol coding]	13–68 [0–68]	39.31 (9.73)	**37.81 (9.61)**	**40.69 (9.65)**

T1: executive functions [experimental data]	Range sample [theoretical]	Total|*M* (*SD*)	Boys|*M* (*SD*)	Girls|*M* (*SD*)
**Inhibition** [interference score—Stroop task]^b^	7.07–89.03 [n.a.]	24.95 (8.78)	**25.70 (9.49)**	**24.26 (8.01)**
**Updating** [correct trials—digit span backwards]	0–13 [0–16]	6.18 (1.47)	6.20 (1.48)	6.16 (1.46)
**Flexibility** [reaction time in milliseconds—feeding fish]^b^	537.26–3299.83 [n.a.]	1658.02 (399.77)	**1695.70 (411.91)**	**1623.47 (385.33)**
**Affective decision-making** [net score—hungry donkey task]	−32 to 50 [−50 to 50]	5.49 (11.43)	**7.10 (12.93)**	**4.01 (9.63)**
**Delay of gratification** [weighted ratio of choices—experimental delay]	0.31–1.0 [0.25–1.0]	0.83 (0.16)	**0.84 (0.16)**	**0.82 (0.16)**

T1–T3: outcomes of EF-profiles [questionnaire data]	Range sample [theoretical]	Total|*M* (*SD*)	Boys|*M* (*SD*)	Girls|*M* (*SD*)
Inhibitory control [TMCQ—parent report]				
T1	1.17–5.00 [1–5]	3.53 (0.67)	**3.44 (0.69)**	**3.61 (0.64)**
T2	1.33–5.00 [1–5]	3.59 (0.63)	**3.51 (0.64)**	**3.66 (0.62)**
T3	1.33–5.00 [1–5]	3.75 (0.68)	**3.68 (0.68)**	**3.82 (0.67)**
Emotional reactivity [BRIEF—parent report]				
T1	1.5–5.0 [1–5]	3.79 (0.71)	3.80 (0.73)	3.79 (0.69)
T2	1.4–5.0 [1–5]	3.86 (0.68)	3.89 (0.70)	3.84 (0.65)
T3	1.2–5.0 [1–5]	3.76 (0.73)	3.79 (0.72)	3.72 (0.74)
Planning/organizing [BRIEF—teacher report]				
T1	1.00–5.00 [1–5]	3.70 (0.89)	**3.50 (0.90)**	**3.89 (0.85)**
T2	1.13–5.00 [1–5]	3.65 (0.90)	**3.41 (0.91)**	**3.87 (0.83)**
T3	1.00–5.00 [1–5]	3.67 (0.96)	**3.48 (0.94)**	**3.86 (0.93)**

Reported range values are sample specific [min/max]; *M*, mean; *SD*, standard deviation; means in bold differ significantly across binary sex (*p* < 0.05).

a Highest reported parental education level.

b Inverted scoring—higher values infer lower EF or SR ability.

Zero-order correlations across all variables are reported in Supplementary Table S3. Age at T1 was significantly positively associated with all five measures of EF at T1. Processing speed showed small positive correlations with inhibition and flexibility (0.20 < *r* > 0.21), but not with the other EF. SES was positively correlated with inhibition, updating, and affective decision-making. The three cool EF were all positively correlated with one another but remained uncorrelated with the two hot EF measures, demonstrating some unity across the cool, but not the hot tasks. Inhibition correlated positively with most SR-outcome measures—most notably with planning and organizing across all time points (T1–T3). Updating also correlated positively with planning and organizing across all time points, and with inhibitory control and emotional reactivity at T1, but not at T2 or T3. The hot EF did not correlate with any of the SR-outcome measures.

### Estimation of latent EF profiles

Using an iterative process, we generated and tested latent models of EF facet performance at T1 with increasing numbers of profiles. Without defining any fixed parameters, a 5-profile model yielded better fit indices (log-likelihood, BIC, AIC), and acceptable profile sizes and entropy compared to a 4-profile model. Although a 6-profile model had even lower fit indices than a 5-profile model, its LMR-adjusted test was non-significant and the 6th class too small to be considered (0.25%) so that the 5-profile model was retained. In a further step, original models were then tested for bivariate within-group residuals across the manifest EF variables (Supplementary Table S4). While the three cool EFs showed residual correlations, the two hot EFs did not. In line with these residuals, conditional independence of the latent models was relaxed, allowing cool EF variables to correlate with one another. The original models were then rerun with this adjustment, thereby changing the fit indices substantially: freeing cool EF correlations led to a stark decrease in the smallest class size for the 5-profile model (0.4% of the sample) and a non-significant LMR-adjusted test. Model fit and entropy for the 4-profile model improved to the extent that it outperformed the original 5-profile model, and it was deemed the best fitting model with entropy = 0.85 and the smallest group size being 4.6% of the sample (*n* = 77) (Table 2 for information on all models). Further model estimations including free variance and/or free hot EF correlation did not improve model fit or did not converge above and beyond 3-profile model solutions and are therefore not reported.

**Table 2:** Latent profile analyses of executive functions—with the 4-profile solution allowing partial conditional dependence providing the best fit.

*N* profiles	*N* free parameters	Log-likelihood	Adjusted BIC^a^	AIC^b^	Entropy	LMR adjusted test^c^ H0 log likelihood	Bootstrapped LRT^d^ H0 log likelihood	Smallest class (% of sample)
1	10	−11401.38	22845.12	22822.77	n.a.	n.a.	n.a.	n.a.
2	16	−11190.31	22448.40	22412.63	0.86	412.86^**^	422.14^***^	12.18
3	22	−10991.97	22147.02	22027.94	0.89	387.96^***^	396.68^***^	7.26
**4**	**28**	**−10901.13**	**22009.83**	**21858.27**	**0.83**	**177.68** ^***^	**181.68** ^***^	**6.88**
**5**	**34**	**−10828.74**	**21909.52**	**21725.48**	**0.79**	**141.60** ^*^	**144.79** ^***^	**3.10**
6	40	−10791.32	21879.15	21662.64	0.81	73.20 (n.s.)	74.84^***^	0.25
	**Relaxing partial conditional independence in models for the correlation of all three cool EF**							
1	13	−11268.45	22591.97	22562.90	n.a.	n.a.	n.a.	n.a.
2	19	−11058.53	22197.54	22155.05	0.94	410.61^***^	419.85^***^	7.80
3	25	−10898.41	21902.72	21846.82	0.92	313.19^*^	320.23^***^	6.04
**4**	**31**	**−10798.82**	**21728.95**	**21659.64**	**0.85**	**194.80** ^*^	**199.18** ^***^	**4.47**
5	37	−10753.81	21664.35	21581.62	0.85	88.04 (n.s.)	90.02^***^	0.40

Correlations across cool EF included: inhibition*updating, updating*flexibility, and flexibility*inhibition. ^*^p < 0.05, ^**^p < 0.01, and ^***^p < 0.001.

a Bayesian information criterion.

b Akaike information criterion.

c Lo–Mendell–Rubin adjusted likelihood ratio test.

d Bootstrapped likelihood ratio test.Fit indices in bold indicate models with good fit.

**Figure 2:**
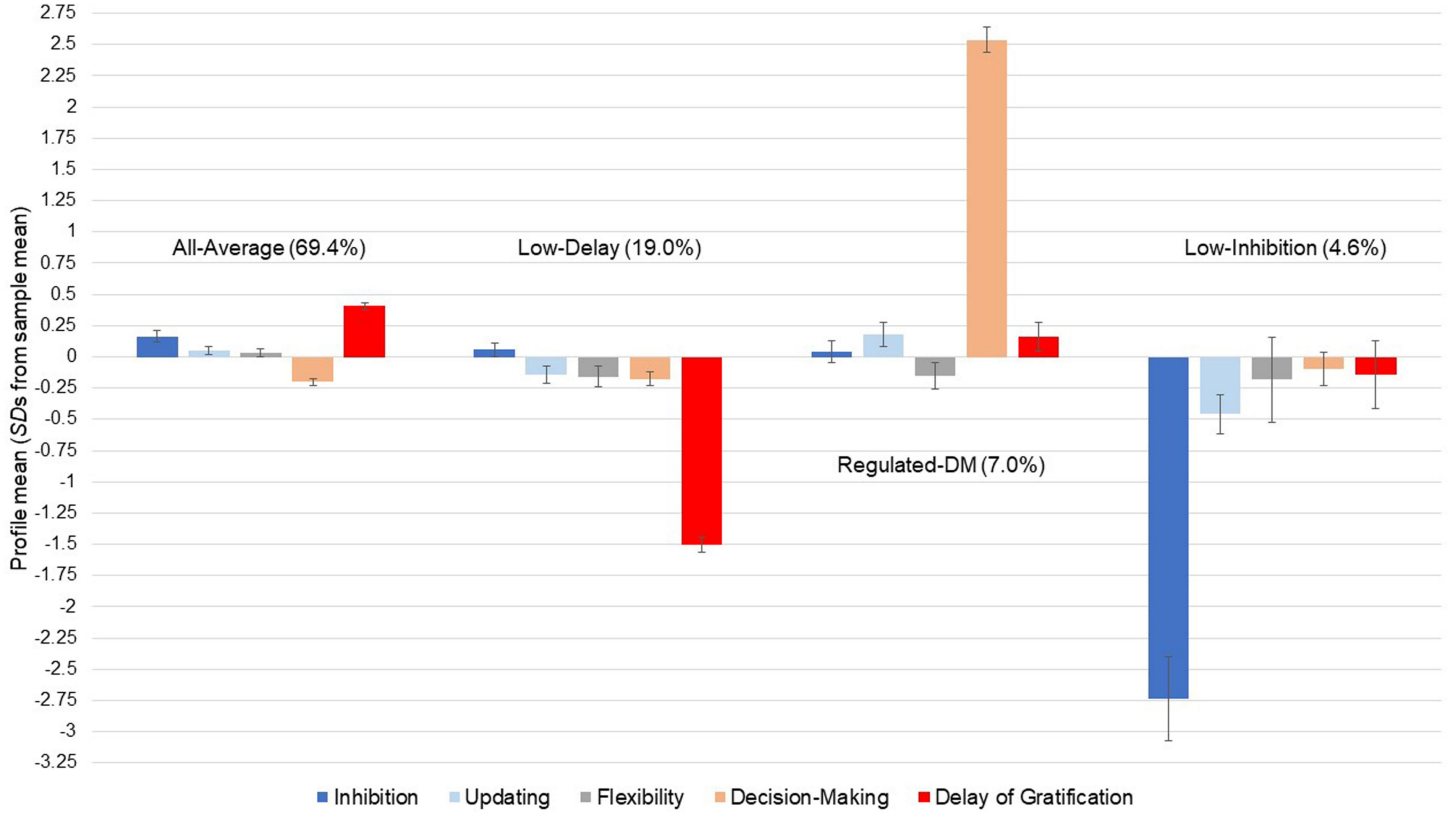
Results of the LPA: mean performance across five executive functions in the latent solution with four profiles assuming partial dependence for cool EF at T1 (age 6 to 11 years; *N* = 1,657; 52.1% female), model entropy = 0.848.

The ensuing analyses were carried out with the 4-profile model assuming partial dependence across the cool EF (i.e., allowing residual correlations). Average latent class probabilities of this model were good and lay between 0.867 and 0.933 across the likely membership—latent class matrix. Figure 2 shows the performance distribution of the *z*-standardized EF facets across the 4 profiles, the absolute values are reported in Table 3. The *all-average* profile was the largest, comprising 69.4% of the sample (*n* = 1,149). Within this group of children, individual performance across the three cool and two hot EF facets at T1 was no more than 0.5 *SD*s above or below the overall sample’s mean in the respective task. The other three profiles were marked by average performance across all EF apart from one facet: the second largest *low-delay* profile included 19.0% of the sample (*n* = 315), with the hot facet delay of gratification averaging 1.5 *SD*s below the sample mean. In the third *regulated-DM* profile (7.0% of the sample, *n* = 116), the hot facet affective decision-making was more than 2.5 *SD*s above the sample mean. These children made more favorable choices, thus displaying regulated DM (rather than affective DM). In the final *low-inhibition* profile (4.6% of the sample, *n* = 77), the cool EF facet inhibition was 2.75 *SD*s below the sample mean. A trend for lower updating is also visible, with children assigned to the *low-inhibition* profile performing at the lower margin, almost 0.5 *SD*s below the sample mean. As predicted (H1), four latent EF profiles of varying performance were identified. However, their shape as determined by mean performances within each profile varied significantly from those initially assumed (H1.1–H1.4), so that our hypothesis on profile prevalence (H2) could not be tested.

**Table 3:** Absolute means for EF facets and characteristics across four EF profiles allowing partial conditional dependence of cool EF.

Mean performance (absolute values, *SD*) across 4 latent EF profiles					
	Sample (100%)	All-average (69.3%)	Low-delay (19.0%)	Regulated-DM (7.0%)	Low-inhibition (4.6%)
**Executive facets**					
Inhibition (I)^a^	24.95 (8.78)	23.52 (0.42)	24.46 (0.52)	24.58 (0.76)	48.97 (2.92)^c^
Updating (U)	6.18 (1.47)	6.25 (0.05)	5.97 (0.10)	6.45 (0.14)	5.51 (0.23)
Flexibility (F)^a^	1658.02 (399.77)	1638.28 (14.72)	1716.92 (33.32)	1714.32 (44.17)	1727.63 (140.44)
Affective DM (ADM)^b^	5.49 (11.43)	3.18 (0.27)	3.49 (0.61)	34.48 (1.17)^c^	4.405 (1.52)
Delay of gratification (D)	0.83 (0.16)	0.90 (0.01)	0.59 (0.01)^c^	0.86 (0.02)	0.81 (0.04)
**Profile characteristics**					
Age [years]	8.36 (0.95)	8.42 (0.93)	8.25 (0.90)	8.47 (0.90)	7.65 (0.88)
Binary sex [%]	52.1	54.1	56.2	29.8	37.8
SES	5.05 (1.00)	5.06 (1.00)	4.99 (1.10)	5.23 (0.86)	4.80 (0.94)
Multilingualism [%]	6.8	4.6	7.1	5.3	12.2
Processing speed [score—digit symbol coding]	39.31 (9.73)	39.44 (9.49)	40.14 (10.57)	38.52 (9.56)	35.27 (9.70)

a Inverted scoring—higher values indicate lower EF or SR ability.

b DM, decision-making.

c ±1.5 *SD*s from sample mean.

### Characteristics of EF profile members

The weighted multinominal logistic regression (Table 4) revealed that age, binary sex, SES, multilingualism, and processing speed at T1 varied significantly across the four identified latent EF profiles. Absolute means of each characteristic across all four latent subgroups can be viewed in Table 3. Although our hypotheses (H3.1–H3.5) referred to other EF profiles, they were mostly confirmed within the 4-profile solution when regarding high vs. low performance. As expected, the only profile marked by above-average (hot) EF performance, *regulated-DM* (7.0% of the sample), was characterized by an increased ratio of children with higher SES (H3.2) than the *low-delay* and *low-inhibition* profiles, and a higher ratio of boys (H3.4b) than the *all-average* and *low-delay* profiles. Contrary to our hypotheses, the *regulated-DM* profile did not differ significantly from the other profiles (except for *low-inhibition*, see below) regarding children’s age (H3.1), processing speed (H3.3), or multilingualism (H3.5).

**Table 4:** Characteristics and demographics associated with each profile in comparison to a reference group as calculated using multinomial logistic regression analyses (log odds [confidence intervals]).

Reference group: all-average (69.3%)	Low-delay (19.0%)	Regulated-DM (7.0%)	Low-inhibition (4.6%)
Age	0.824 [0.707–0.959]^*^	1.033 [0.846–1.261]	0.336 [0.246–0.460]^***^
Binary sex	1.065 [0.814–1.393]	0.359 [0.233–0.554]^***^	0.560 [0.333–0.939]^*^
SES	0.902 [0.772–1.054]	1.213 [0.977–1.506]	0.688 [0.543–0.872]^**^
Multilingualism	1.602 [0.927–2.767]	1.187 [0.489–2.881]	2.636 [1.183–5.876]^*^
Processing speed	1.033 [0.898–1.189]	0.970 [0.788–1.193]	0.574 [0.442–0.746]^***^
Reference group: low-delay (19.0%)		Regulated-DM (7.0%)	Low-inhibition (4.6%)
Age		1.255 [0.993–1.585]	0.408 [0.292–0.570]^*^
Binary sex		0.338 [0.209–0.546]^***^	0.526 [0.302–0.915]^***^
SES		1.344 [1.047–1.727]^*^	0.763 [0.585–0.994]^*^
Multilingualism		0.741 [0.282–1.947]	1.646 [0.679–3.991]
Processing speed		0.938 [0.741–1.187]	0.556 [0.419–0.737]^***^
Reference group: regulated-DM (7.0%)			Low-inhibition (4.6%)
Age			0.325 [0.227–0.467]^***^
Binary sex			1.557 [0.811–2.988]
SES			0.567 [0.418–0.769]^***^
Multilingualism			2.220 [0.703–7.015]
Processing speed			0.592 [0.429–0.817]^**^

Binary sex was coded 0 for boys and 1 for girls; Multilingualism was coded 0 for monolingual and 1 for multilingual children. ^*^p < 0.05, ^**^p < 0.01, ^***^p < 0.001.

The second-largest profile, *low-delay* (19.0% of the sample) included a higher ratio of younger children (H3.1) than the *all-average* profile. The significant log odds in reference to the profiles *regulated-DM* (binary sex, SES; see above) and *low-inhibition* (age, binary sex, SES, processing speed; see below) also confirmed our hypotheses. The *low-delay* profile did not differ significantly from any other profile regarding multilingualism, but because (H3.5) referred to deviant cool EF performance, this hypothesis was not relevant for this (or for the *regulated-DM*) profile.

The profile marked by below-average cool EF performance, *low-inhibition* (4.6% of the sample), differed not only from *low-delay*, but also from the other profiles, and in all five analyzed characteristics. Consistent with our hypotheses, *low-inhibition* was characterized by a higher ratio of younger children (H3.1), lower SES (H3.2), and lower processing speed (H3.3) than all other profiles, as well as a higher ratio of boys (H3.4a) than *all-average* and *low-delay*. Contrary to our assumptions, *low-inhibition* included a higher (not lower; H3.5) ratio of multilingual children than *all-average*. Belonging to this subgroup marked by underperformance in behavioral inhibition and trending underperformance in working-memory updating was thus associated with significantly lower or compromised values across an entire set of characteristics that are potential risk-factors for childhood EF development.

### Predictive value of EF profile membership for later SR outcomes

Within hierarchical linear regression analyses, the weighted profile membership at T1 was only included as a predictor if prior bivariate correlations with the respective outcome variable of SR at T2 and T3 (ca. 1 year and 3 years after T1) were significant (see Supplementary Table S5). Consequently, the *low-inhibition* profile was used most frequently, and the *low-delay* profile was not used at all within the regression analyses (Table 5). In line with our hypothesis (H4.1), inhibitory control was predicted to a certain extent: a higher probability of belonging to the *low-inhibition* profile significantly predicted lower inhibitory control at T2 above and beyond inhibitory control at T1, *F*(2, 1,086) = 411.81, *p* < 0.001; 43.1% of the variance explained. However, belonging to the *low-inhibition* profile did not predict inhibitory control at T3. Unexpectedly, the ability to plan and organize at T2 was not significantly predicted by T1 EF profile membership above and beyond its original rate at T1. Unlike hypothesized (H4.2), a higher probability of belonging to the *regulated-DM* profile significantly predicted lower (not higher) planning/organizing at T3, above and beyond its expressions at T1 and T2, *F*(3, 850) = 194.85, *p* < 0.001; 40.7% of the variance explained. Contrary to (H4.3), emotional reactivity was not predicted by profile membership from T1 to T2 or T3.

**Table 5:** Hierarchical linear regressions of complex SR outcomes at T2 and T3 according to profile membership probability.

Independent variables	*R* ^2^	Δ*R*^2^	*F*^2^ change	*β*	Beta	*T*
**Regressing inhibitory control at T2 on EF-profile membership**						
**Step 1:** inhibitory control T1	0.428	0.428	814.429^***^	0.621	0.654	28.538^***^
**Step 2:** inhibitory control T1	0.431	0.003	5.680^*^	0.617	0.651	28.355^***^
*Low-inhibition* profile				−0.187	−0.055	−2.383^*^
**Regressing inhibitory control at T3 on EF-profile membership**						
**Step 1:** inhibitory control T1	*0.*422	0.422	346.119^***^	0.297	0.292	8.936^***^
Inhibitory control T2				0.450	0.420	12.862^***^
**Step 2:** inhibitory control T1	0.422	0.000	0.001	0.297	0.292	8.931^***^
Inhibitory control T2				0.450	0.420	12.820^***^
*Low-inhibition* profile				−0.003	−0.001	−0.035
**Regressing emotional reactivity at T2 on EF-profile membership**						
**Step 1:** emotional reactivity T1	0.488	0.488	1040.189^***^	0.664	0.698	32.252^***^
**Step 2:** emotional reactivity T1	0.488	0.000	0.960	0.663	0.697	32.159^***^
*Low-inhibition* profile				−0.077	−0.021	−0.980
**Regressing planning/organizing at T2 on EF-profile membership**						
**Step 1:** planning/organizing T1	0.651	0.651	2117.244^**^	0.814	0.807	46.014^***^
**Step 2:** planning/organizing T1	0.652	0.001	1.543	0.810	0.803	45.238^***^
*Low-inhibition* profile				−0.159	−0.033	−1.740
*All-average* profile				−0.015	−0.006	−0.346
**Regressing planning/organizing at T3 on EF-profile membership**						
**Step 1:** planning/organizing T2	0.404	0.404	288.787^***^	0.281	0.262	5.849^***^
Planning/organizing T2				0.431	0.405	9.051^***^
**Step 2:** planning/organizing T1	0.408	0.003	1.641	0.284	0.265	5.910^***^
Planning/organizing T2				0.430	0.404	9.025^***^
*Low-inhibition* profile				0.026	0.005	0.170
*Regulated-dm* profile				−0.257	−0.063	−2.068^*^
*All-average* profile				−0.037	−0.015	−0.474

Regressions were only calculated with respective profile probability variables if these correlated with outcome variables. Therefore, each regression includes a unique set of profile membership variables. Because no profile probability variable correlated with emotional reactivity at T3, this regression was omitted. ^*^p < 0.05, ^**^p < 0.01 and ^***^p < 0.001.

## Discussion

By identifying four latent EF profiles with varying manifestations of three cool and two hot EF facets, we demonstrated the inter-and intraindividual variation (between-subject differences across persons and between-facet differences within persons, respectively) and heterogeneity of a comprehensive set of EF within a large elementary-school aged community sample (*N* = 1,657; age 6–11 years at T1). Our initial predictions were mainly based on results of variable-oriented research, due to a lack of prior person-oriented approaches within the field. This may explain why the identified EF profiles at T1 did not manifest as predicted: while our hypotheses regarding profile performance were *factor*-oriented (i.e., cool vs. hot), results redirected us to *facet*-oriented profile performance characteristics and conclusions. Although these profiles were unexpected, a high entropy and strong latent class probability matrices supported the statistical strength of the model: the most prevalent *all-average* profile (69% of the sample) can be interpreted as a baseline pattern for “normative” and age-appropriate EF performance. A large proportion of children deviated from this norm in at least one EF facet. The *low-delay* (19%), *regulated-DM* (7%), and *low-inhibition* (5%) profiles were characterized by above-or below-average performance in singular EF facets, with average performance in the other investigated facets. This suggests that the cool/hot factor structure of EF, which is prominent in most variable-oriented research within developmental cognition (Best and Miller, 2010; Brydges et al., 2014; Harms et al., 2014; Fernández García et al., 2021), does not play a fixed role when viewed from a person-oriented perspective. The present *all-average* and *low-inhibition* profiles replicate similar profiles identified by Chaku et al. (2022) in a slightly older population in one of the few other LPA analyses, supporting our findings’ plausibility. The two remaining profiles *low-delay* and *regulated-DM* add novel insight to the field, via the integration of two hot facets into the latent analyses. Additionally, children belonging to different EF profiles showed significant variations in demographic characteristics including age, binary sex, SES, multilingualism, and processing speed. Regarding the predictive value of EF profile membership, we found that profile membership at T1 was associated with some of the later SR-outcomes at T2 or T3 (i.e., 1 or 3 years later). This underlines the between-subject and between-facet heterogeneity of self-regulation and its required basal cognitive and behavioral functionality during childhood development. Our person-oriented analyses thus provide a new perspective on individual differences in the simultaneous expression of a comprehensive set of EF facets in middle childhood.

### EF profiles and concurrent characteristics

The 6- to 11-year-olds in the most prevalent *all-average* profile displayed representative EF performance across all five EF facets and were characterized by average to higher SES and processing speed compared to children belonging to other profiles. *All-average* profile membership did not significantly predict SR outcomes 1 or 3 years later. The second most prevalent *low-delay* profile included children who preferred immediate gratification in the form of small gifts over long-term reward. These children were significantly younger than *all-average* children and more frequently female compared to children in the *regulated-DM* and *low-inhibition* groups. Children in the *regulated-DM* profile excelled at decision-making. They made more favorable choices in the age-appropriate version of the Iowa gambling task, resulting in lower unpredictable losses and therefore in long-term advantages. As expected, these children were frequently male and had a higher SES than children belonging to the two low performance groups.

The two identified hot-EF profiles did not manifest in the form of *high-cool, low-hot EF* as expected. Instead, the subgroups showed above-/below-average performance in only one but not the other hot EF task. The identified *low-delay* and *regulated-DM* profiles suggest that at elementary-school age, the variable-oriented link between hot facets is not (yet) evident in latent profiles, and that expressions of hot EF facets are independent from one another. This in line with other work (Hongwanishkul et al., 2005; Brydges et al., 2014; O’Toole et al., 2018) suggesting that in 3- to 6-year-olds, cool EF were substantially positively intercorrelated, but hot EF were not or negatively intercorrelated after controlling for confounding variables. We extended these observations to an older cohort, evident in both non-significant zero-order correlations between hot EF and also in their independence during latent modeling. Our person-oriented analyses thus indicate that correlations between affective decision-making and delay of gratification (as measured in the present tasks) may not develop until early adolescence. Of note, *low-delay* (i.e., displaying a preference for an immediate smaller reward) was more frequent for girls than boys, and *regulated-DM* rather than affective DM (i.e., oriented towards lower immediate reward, but long-term advantage) was observed more frequently for boys than girls. Typically, adult males and teenage boys tend to show riskier behavior than female participants in the Iowa gambling task or its age-appropriate versions (Blakemore and Choudhury, 2006; Orm et al., 2022). This sex difference is not yet visible in our sample spanning middle childhood, but might develop later (Poon, 2017; Orm et al., 2022), potentially due to differences in the maturation speed of the orbital prefrontal cortex (Lensing and Elsner, 2018).

The least prevalent yet highly interesting *low-inhibition* profile (5%, *n* = 77) was marked by higher interference in the incongruent condition of the fruit-/vegetable-stroop task, indicating problems when inhibiting a prepotent response. These children also showed a trend to lower working-memory updating in the digit span backwards task. The profile confirms a similar profile found by Chaku et al. (2022) in a slightly older sample, which counters the notion that the younger age and higher ratio of boys within this *low-inhibition* profile might simply depict an earlier stage of (cool) EF manifestation (Hooper et al., 2004) within our sample. Inhibition is frequently mentioned as one of the first distinct EF facets to develop, but sometimes also as an underlying “general executive” with vast explanatory power (Boelema et al., 2014; Friedman and Miyake, 2017; Hartung et al., 2020). Significant deficits in inhibition during early and middle childhood have therefore been associated with problematic characteristics and future challenges. In line, children in the *low-inhibition* profile had the lowest average SES, and higher odds for speaking more than one language compared to others, suggesting a more frequent upbringing in a multicultural setting for some of these individuals. The combination of low SES and slower processing speed might mark the *low-inhibition* profile as a risk profile of children with less resources and privileges than their peers. This, in turn, could reflect poorer integration in school and reduced advancement in scholastic and executive ability. One might assume that the prevalence of certain profiles is dependent on the sample demographics: the *low-inhibition* profile might become more prevalent in a sample with a lower SES. This assumption would parallel a prior LPA carried out in preschool-aged children from low-income communities, in which a *low-EF* profile had a high prevalence of 52% (Williams and Bentley, 2021). Additionally, the *low-inhibition* profile may potentially correlate with ADHD, as observed frequently in boys with poor inhibitory control (Dajani et al., 2016; Jacobson et al., 2018). This association, along with the profiles’ higher prevalence in a lower SES setting could qualify the profile as a valuable risk marker for attentional or academic issues.

The presented 4-profile structure was selected based on several evaluation criteria. To determine the best fitting latent profile model, variations in model restriction were examined and cool EF residual correlations were ultimately allowed to improve model fit. This indicates that associations across cool EF were not fully explained by initial profile modeling, aligning with the notion that cool EF reliably correlate in early childhood as evident within the current sample of 6- to 11-year-olds (Zelazo and Carlson, 2012; Welsh and Peterson, 2014). Of note, an alternative model that assumed variable independence across all five EF (see Supplementary Figure S2 and Supplementary Table S6) yielded some shifts within the four reported profiles. Most evident was a picture of more accentuated below-average performance on all three cool EF facets in the so-far described *low-inhibition* profile, as well as an increased prevalence (from 5% up to 9%). Hence, if cool EF are not allowed to correlate during modeling, one could rename the resulting profile *low-cool EF* because of a significant decrease in performance for updating and flexibility. This trend towards a more integrative *low-cool* instead of *low-inhibition* profile may become more distinct in a slightly younger age group or in a slightly larger subgroup of at-risk children. This, in turn, may provide more insight into the initial high intercorrelation and progressive differentiation of a cool EF factor across childhood (Lee et al., 2013). Our results also contribute to the ongoing discussion regarding the dimensionality of the “temperature” of EF facets (Moriguchi and Phillips, 2023). While there seems to be a clear theoretical dissociation between more rational (cool) and more emotional (hot) EF facets, the temperature of these abilities is highly dependent on the complexity of the situation in which the EF are applied, including aspects of emotion and motivation, as well as the type of experimental tasks used to measure the EF (Peterson and Welsh, 2014). This contextual and methodological framework should always be kept in mind when extrapolating results from experimental paradigms to more ambiguous real-life settings.

Of interest, our LPA did not yield an EF-profile marked by *high inhibition* or *high inhibition and updating* (H1.4). Apparently, children with higher cool EF were subsumed within the large group of *all-average* children within our sample, and further dividing this subgroup did not increase the model’s explanatory and statistical power. This provides support for the assumption that the differentiation of a stronger cool EF factor during middle childhood is not a defining influence on profile generation, and contrasts our original assumption which was based mainly on variable-oriented studies (Hughes et al., 2010; Peterson and Welsh, 2014). Following up on the stability and discontinuity of the reported EF profiles may provide further insights into the longitudinal intraindividual developmental correlations of the cool/hot EF factors and EF facets across adolescence, which may lead to changes in profile shapes and to transitions of individuals between the EF profiles.

### Predictions by EF profiles on consequent SR outcomes

We assumed that profile membership at T1 would predict parent- and teacher-reported SR outcomes for inhibitory control, planning/organizing abilities, and emotional reactivity at T2 and T3 (approximately 1 and 3 years later). However, this only held true for two EF profiles and two SR outcomes: parent-reported inhibitory control at T2 (but not at T3) was negatively predicted by the likelihood of belonging to the *low-inhibition* profile at T1. Thus, elementary school-aged children with lower basal inhibitory abilities and concurrent characteristics including lower SES, lower processing speed, and a higher likelihood of growing up in a multilingual environment seem to be at increased risk of future impediments in daily inhibitory control compared to peers. Considering the abundant evidence showing a positive relation between cool EF ability and educational and occupational performance (Poon, 2017; Robson et al., 2020), it follows that a lower ability to inhibit intruding or dominant behaviors may contribute to a lack in perseverance when executing important tasks. This may be of specific relevance when transitioning into elementary school. Given the construct-overlap of basal, behavioral inhibition and more situational inhibitory control, we assume that these trends will magnify in a larger group of *low-inhibition* individuals, which in turn might also lead to an extension of the negative effects across a protracted period of time.

An unexpected finding of our analyses was that the teacher-reported ability to plan and organize at T3 was negatively predicted by the likelihood of belonging to the *regulated-DM* profile. That is, children who opted for relatively more favorable and less risky choices at T1 had significantly more issues structuring and organizing their daily tasks, chores, and assignments 3 years later (even when controlling for their planning/organizing status at T1 and T2). One explanation may be that the negative association displays the dissociation between motivational versus rational contexts: making cautious decisions about “hot” reward and loss contingencies was negatively associated with making “cool” plans and decisions later on. As discussed above, more research is needed to confirm this insight into developmental EF and SR associations provided by the current person-oriented approach.

Contrary to our expectations, parent-reported emotional reactivity at T2 or T3 was not significantly predicted by T1 profile membership. The lacking long-term association may be because emotional reactivity is an early marker of temperament with substantial stability from early childhood onwards, rather than being influenced by protracted EF development (Ursache et al., 2013). Additionally, SR and EF measured experimentally often show little overlap with accordant parent reports, potentially due to a dissociation between experimental tasks and the daily situations and problems observed by parents (Röthlisberger et al., 2010; Robson et al., 2020). While the lack of associations between the identified EF profiles and many reported SR outcomes was unexpected, stronger prospective links might be seen if self-reported or behavioral SR outcomes were added to the design.

### Limitations

Despite the large sample size and sophisticated person-oriented statistical approach, this investigation is not without limitations. The large community-based sample of elementary school-aged children in Germany supports the generalizability of our findings regarding EF performance to other similar populations in western culture. Based on the association of our findings with demographic variables (including SES and multilingualism), we acknowledge that similar analyses may yield different findings in populations originating from other cultures or socio-economic backgrounds. These potential discrepancies should be examined and discussed in future research.

Missing data and drop-outs for parent-and teacher-reported SR outcomes were relatively high. Reasons for their missing contribution could include a lack of resources, health issues, or their child’s problem behavior. Therefore, our findings regarding the potential SR outcomes (T2–T3) of profile membership should be interpreted with caution. The use of experimental data for the identification of EF-profiles versus other-reported data for the investigation of potential SR outcomes may explain the apparent lack of the profiles’ predictive power. Studies and meta-analyses have indicated that differing measurement modalities often result in reduced synthesis—even when these modalities attempt to measure the same theoretical constructs (Wallisch et al., 2018; Eisenberg et al., 2019). This is also linked to a state-trait disparity that is seldom addressed in research on EF development. While many studies assume EF to be a stable trait based partially on biological factors (Engelhardt et al., 2015), others indicate fluctuations and states of EF in repeated measurements (Ludwig et al., 2016) for example due to a child’s level of exercise, affective state, or quality of social interaction before testing (Becker et al., 2014; Ludwig et al., 2016; Moschko et al., 2022). Thus, experimental paradigms may capture more of the current SR state, whereas parent- or teacher-reported data captures more general patterns of SR.

Due to time constraints during data collection, we used some measures in shortened formats. This may have compromised their reliability and validity to a certain extent, despite careful theoretical and statistical considerations. In particular, the hungry donkey task operationalizing affective decision-making, which consisted of 60 trials instead of the original 200 trials may have been affected (Crone and van der Molen, 2004). As demonstrated by Cortes-Patino et al. (2017) in a sample aged 8–17 years, these extra trials may be crucial for children to really apprehend the reward contingencies associated with each door. In their study, only 36% of the sample reached choice-stability for advantageous doors by the 200th trial. Fittingly, previous analyses of the present data at T1 found a significant increase of the hungry donkey net scores (indicating a shift towards favorable decisions) across three 20-trial blocks with no ceiling effect (Lensing and Elsner, 2017), implying that the 6- to 11-year-olds in our sample may not have reached the peak of their learning curve in the shorter format. These observations underline how challenging it can be to measure EF facets in younger samples both efficiently and accurately.

Lastly, while several characteristics of EF profile membership at T1 were identified, further markers of membership surely exist. Verbal and reading abilities have been shown to influence both the baseline ability and growth trajectory of cool EF across 4- to 6-year-old children (Hughes et al., 2010; Berthelsen et al., 2017). Evidence from twin and individual studies also demonstrates that EF are highly heritable and have strong genetic influences (Engelhardt et al., 2015; Li et al., 2015; Friedman et al., 2016).

### Outlook

This study provides important insights on the concurrent intra- and interindividual differences in EF performance during middle childhood. Through a person-oriented approach, we provide a new perspective on the expression and differentiation of three cool EF facets (inhibition, working-memory updating, cognitive flexibility) and two hot EF facets (affective decision-making, delay of gratification) during an important period of cognitive and social development. The four identified profiles support the idea that EF facet development might entail quite individual performance manifestations, with stronger intercorrelations among the cool EF facets. The profiles did not align with the assumption derived from prevailing variable-oriented research that cool, basal EF would dominate in the studied age range via high-performance profiles. Instead, a profile marked by significant below-average performance in inhibition was identified and was associated with lower SES, and lower processing speed, a higher prevalence among boys and children from multilingual families. The profile additionally predicted significantly lower measures of inhibitory control 1 year later, marking the *low-inhibition* profile as a potential risk-profile. As proposed by Engelhardt et al. (2015), early cool EF expression may be considered a developmental endophenotype for later cognitive abilities, strengths and weaknesses. The present study extends on this proposal by suggesting that certain profiles including both cool and hot EF may be viewed as meaningful endophenotypes and risk factors for SR abilities. Taken together, the identified EF profiles in 6- to 11-year-olds should make us question whether there is solely one trajectory of EF development or whether normative children can undergo varying routes and cascades of development, depending on the children’s prerequisites, existing risk factors, or perhaps even genetic assets. Future studies might attempt to replicate these EF profiles and follow-up on their developmental trajectories and associations with various outcome measures to extend on a person-oriented understanding of the underlying dynamics. Likewise, considering such profiles of EF performance within application-oriented research may help devise increasingly targeted, personalized, and efficient education strategies and cognitive intervention programs. This, in turn, may help personalize schooling approaches in order to foster a balanced individual differentiation of all facets of executive functioning for the varying developmental profiles of different children over time.

## Data availability statement

The datasets presented in this article are not readily available because the raw data supporting the conclusions of this article are part of a larger longitudinal study with strict data protection guidelines. Upon completion of the ongoing data collection and publications, data will be made available in a public repository. Requests to access the datasets should be directed to AB, ariadne.brandt.1@uni-potsdam.de.

## Ethics statement

The studies involving humans were approved by the Research Ethics Board at the University of Potsdam as well as the Ministry of Education of Brandenburg, Germany. The studies were conducted in accordance with the local legislation and institutional requirements. Written informed consent for participation in this study was provided by the participants’ legal guardians/next of kin.

## Author contributions

AB: Conceptualization, Formal analysis, Investigation, Writing – original draft, Writing – review & editing. RB: Conceptualization, Funding acquisition, Writing – review & editing. BE: Conceptualization, Funding acquisition, Supervision, Writing – review & editing.

## Footnotes

^1^The SR outcome anger regulation (Grob and Smolenski, 2005) was initially preregistered but excluded due to weak factor solutions within the sample. Details are provided in the Supplementary Table S1.
