# Supplementary material for: Profiles of executive functions in middle childhood and prediction of later self-regulation
Source: Front Psychol. 2024 May 9;15:1379126. doi: 10.3389/fpsyg.2024.1379126 (PMC11111993; doi:10.3389/fpsyg.2024.1379126)
Supplement: Supplementary file 1 [file Data_Sheet_1.pdf]

Supplementary Material

Profiles of Executive Functions in Middle Childhood and Prediction of Later Self-Regulation

Ariadne Brandt, Rebecca Bondü, and Birgit Elsner

Table of Contents

Table S1 Exploratory Factor Analysis for Items on the FEEL-KJ Questionnaire Operationalizing Anger Regulation (excluded from the present study) ………………………2

Table S2 Fit Indices of Latent Profile Analyses of Executive Functions for Younger (6 - 7.99 Years) and Older (8 - 9.99 Years) Age Groups…………………………………………………..….4

Figure S1 Mean Performance Across Executive Functions for Younger (6 – 7.99 Years) and Older (8 – 9.99 Years) Age Groups in the LPA Solution with Four Profiles Assuming Partial Dependence for Cool EF……………………………………………………………….....5

Table S3 Zero-Order Correlations Across all Variables of Interest (Spearman Rho)……….....6

Table S4 Correlations of Residuals for Latent Profile Model Solution with Four Profiles..….8

Table S5 Bivariate Correlations of Profile Membership Probabilities (Gained from Latent Profile Analysis) and Self-Regulatory Outcomes…………………………………………...9

Figure S2 Mean Performance Across Five Executive Functions in Four Latent Profiles Assuming Partial Conditional Independence Across All Variables Within a Healthy Sample of Children in Elementary School (N = 1,657), Model Entropy = .827…………………………10

Table S6 Z-Standardized Means for Latent EF Profile Model Including Four Profiles and Assuming Partial Conditional Independence of All Variables………………………………10

References……………………………………………………………………………11

Table S1

Exploratory Factor Analysis for Items on the FEEL-KJ Questionnaire Operationalizing Anger Regulation (excluded from the present study)

Items	Loadings	α	Eigenvalues	Total % of variance	KMO[a]
**Self-reported anger regulation T1**		.217	1.18	11.00	.523
Item 1	.084				
Item 2	.026				
Item 3	.219				
**Self-reported anger regulation T2**		.326	1.28	15.39	.553
Item 1	.095				
Item 2	.273				
Item 3	.095				
**Self-reported anger regulation T3**		.375	1.35	22.93	.522
Item 1	.033				
Item 2	.483				
Item 3	.172				
**Parent-reported anger regulation T1**		.311	1.27	15.56	.547
Item 1	.073				
Item 2	.097				
Item 3	.297				
**Parent-reported anger regulation T2**		.236	1.19	10.22	.537
Item 1	.046				
Item 2	.112				
Item 3	.148				
**Parent-reported anger regulation T3**		.350	1.36	22.49	.518
Item 1	.020				
Item 2	.399				
Item 3	.256				

[a]Kaiser-Meyer-Olkin Criteria

Anger regulation was operationalized using a subset of items from the ‘Fragebogen zur Erhebung der Emotionsregulation bei Kindern und Jugendlichen’ (FEEL-KJ; Questionnaire for Assessing Emotion Regulation in Children and Adolescents; Grob and Smolenski, 2005). The items are conceptualized to capture different behaviors and strategies used by children as they navigate their feelings (i.e. ‘I do something fun to distract myself’). Exploratory factor analysis for three time points and two reporters (children and parents) of anger regulation using the FEEL-KJ questionnaire was carried out, prior to modelling. Four self-reported items originating from different subscales of the questionnaire were presented to children, who replied on a 3-point Likert scale ranging from 1 (*never*) to 3 (*often*). Six items, reformulated for parent-report, were presented to parents using a 5-point Likert scale ranging from 1 (*never*) to 5 (*always*). In line with Rohlf and Krahé (2015), we attempted to combine three reoccurring and theoretically aligned items from each report into one factor each for

further analysis. However, the exploratory factor analysis indicated that items within each report seldom correlated strongly enough ($r > .3$) to construct valid factors. Additionally, the Kaiser-Meyer-Olkin Test did not exceed .6 for groupings, and the explained variance for a one-factor solution frequently fell short of 20%. We thus deemed the factor solution too weak for further consideration and excluded this variable from modelling.

Table S2

Fit Indices of Latent Profile Analyses of Executive Functions for Younger (6 - 7.99 Years) and Older (8 - 9.99 Years) Age Groups

N profiles	*N* free parameters	Log-likelihood	Adjusted BIC[a]	AIC[b]	Entropy	LMR adjusted Test[c] H0 log likelihood	Bootstrapped LRT[d] H0 log likelihood	Smallest class (% of sample)
**Younger Age Group (6 – 7.99 years, *n* = 624)**								
2	19	-4196.56	8455.09	8431.12	.91	115.23 (n.s.)	118.22***	8.15
3	25	-4138.82	8359.18	8327.65	.91	112.56***	115.47***	6.12
**4**	**31**	**-4100.89**	**8302.88**	**8263.78**	**.84**	**73.95****	**75.87*****	**5.88**
5	37	-4074.00	8268.67	8222.00	.84	52.42 (n.s.)	53.78***	.48
6	43	-4058.19	8256.62	8202.39	.86	30.81 (n.s.)	31.61***	.49
**Older Age Group (8 - 9.99 years, *n* = 984)**								
2	19	-6615.12	13301.00	13268.40	.93	283.67***	290.53***	9.26
3	25	-6535.72	13164.32	13121.43	.92	155.22*	158.97***	5.87
**4**	**31**	**-6479.52**	**13074.23**	**13021.05**	**.85**	**109.73****	**112.38*****	**4.11**
5	37	-6458.39	13054.26	12990.79	.87	41.27***	42.26***	1.23
6	43	-6434.64	13029.05	12955.28	.84	46.39*	47.51***	1.37

Note. Partial dependence across cool EF included: Inhibition*Updating, Updating*Flexibility, and Flexibility*Inhibition
[a]Bayesian information criterion, [b]Akaike information criterion, [c]Lo-Mendell-Rubin adjusted likelihood ratio test, [d]Bootstrapped likelihood ratio test,
*$p < .05$, **$p < .01$, *** $p < .001$

Figure S1

Mean Performance Across Executive Functions for Younger (6 - 7.99 Years) and Older (8 – 9.99 Years) Age Groups in the LPA Solution with Four Profiles Assuming Partial Dependence for Cool EF

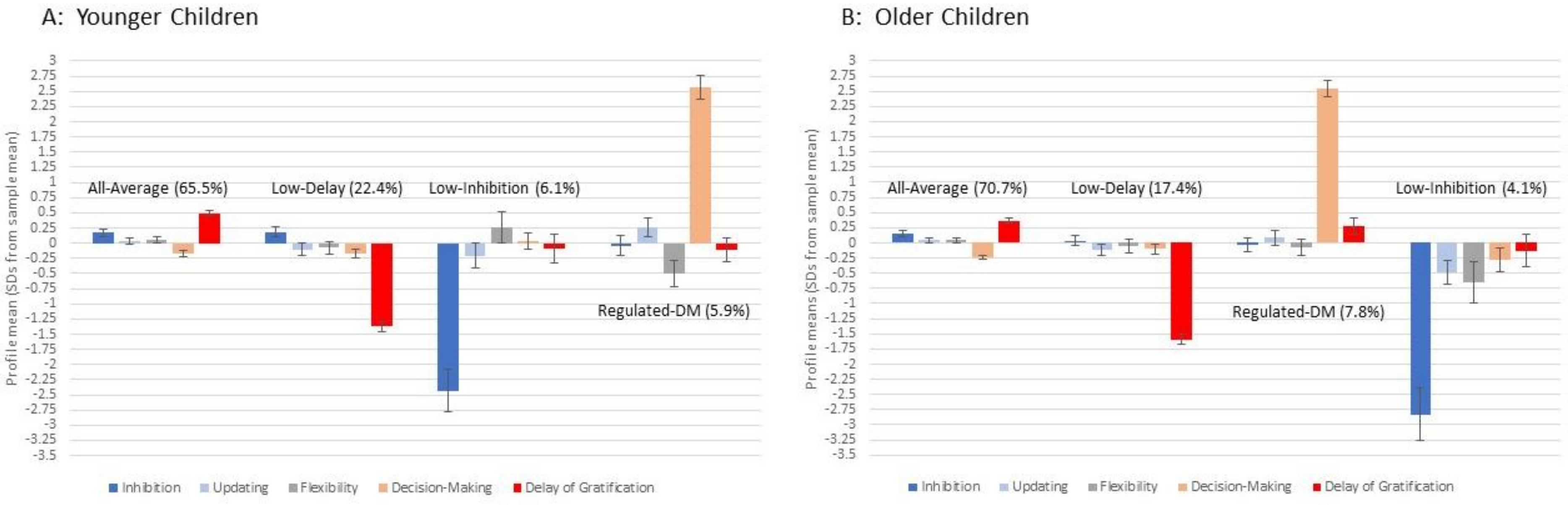

For both the younger (*n* = 624) and older (*n* = 984) age group, a latent profile model including four profiles and partial cool dependence provided the best fit. Small variations in profile prevalences can be noted between the younger and older age group. The *low-inhibition* profile was more prevalent for the younger cohort than the *regulated-DM* profile than for the older children. The older children belonging to the *low-inhibition* profile also showed poorer cognitive flexibility (more than 0.5 SDs from the subsample mean), which was not the case for younger children. Nevertheless, differences were slight. In favor of simplicity and statistical power, the profiles for the entire sample (*N* = 1,657) and age as a significant characteristic were reported in the study.

Table S3

Zero-Order Correlations Across all Variables of Interest (Spearman Rho)

	1	2	3	4	5	6	7	8	9
1. Age at T1	-								
2. Processing speed	-.141**	-							
3. Multilingual background	-.065	-.008	-						
4. Socio-economic background	-.054	.023	.011	-					
5. Inhibition[a]	.355**	.205**	-.034	.081*	-				
6. Updating	.238**	.050	-.074*	.174**	.282**	-			
7. Flexibility[a]	.308**	.201**	.034	.006	.339**	.116**	-		
8. Affective decision-making	.064*	-.008	-.001	.057*	.043	.064*	.004	-	
9. Delay of gratification	.071*	-.009	-.079*	.046	.029	.058	.022	.021	-
10. Inhibitory control T1	.052	.038	.022	.173**	.096**	.081*	.043	.013	.018
11. Inhibitory control T2	.046	.003	-.010	.139**	.082*	.047	.021	.018	-.008
12. Inhibitory control T3	.050	.040	-.034	.173**	.091*	.054	.043	.045	.034
13. Emotional reactivity T1	-.015	.056	-.003	.028	.085*	.097**	.030	.041	.030
14. Emotional reactivity T2	-.005	.034	-.016	.047	.098**	.052	.005	.061	.032
15. Emotional reactivity T3	.018	.005	-.021	.040	.067	.049	.021	.048	.002
16. Planning / organizing T1	.029	.246**	-.024	.230**	.280**	.264**	.159**	.016	.041
17. Planning / organizing T2	.009	.227**	-.013	.216**	.278**	.239**	.153**	.003	-.012
18. Planning / organizing T3	-.078*	.201**	-.022	.241**	.223**	.177**	.058	-.013	.000

	10	11	12	13	14	15	16	17
10. Inhibitory control T1	-							
11. Inhibitory control T2	.639**	-						
12. Inhibitory control T3	.559**	.600**	-					
13. Emotional control T1	.377**	.277**	.206**	-				
14. Emotional control T2	.329**	.337**	.274**	.686**	-			
15. Emotional control T3	.294**	.279**	.309**	.609**	.639**	-		
16. Planning / organizing T1	.285**	.265**	.267**	.129**	.097*	.096*	-	
17. Planning / organizing T2	.284**	.249**	.256**	.151**	.091*	.134**	.809**	-
18. Planning / organizing T3	.237**	.212**	.264**	.185**	.168**	.194**	.587**	.617**

Note. Pairwise deletion of missing data, resulting in a range of n = 750 – 1,647, depending on variable modality (behavioral, parental or teacher report) and time point of collection (T1 – T3)

[a]Inverted so that higher scores infer higher EF facilities for easier readability and comparisons

*p < .01. **p < .001

Table S4

Correlations of Residuals for Latent Profile Model Solution with Four Profiles

**Class 1 (*regulated-DM* profile)**					
	**I**	**U**	**F**	**ADM**	**D**
Inhibition	-				
Updating	-.027	-			
Flexibility	-.157	-.157	-		
Affective decision-making	-.022	.088	-.014	-	
Delay of gratification	.323	-.033	-.105	-.025	-
**Class 2 (*low-delay* profile)**					
	**I**	**U**	**F**	**ADM**	**D**
Inhibition	-				
Updating	.245	-			
Flexibility	.145	.047	-		
Affective decision-making	.046	.029	.015	-	
Delay of gratification	.055	-.098	.025	-.004	-
**Class 3 (*all-average* profile)**					
	**I**	**U**	**F**	**ADM**	**D**
Inhibition	-				
Updating	.244	-			
Flexibility	.269	.105	-		
Affective decision-making	.067	.067	.024	-	
Delay of gratification	-.044	-.016	-.037	.007	-
**Class 4 (*low-inhibition* profile)**					
	**I**	**U**	**F**	**ADM**	**D**
Inhibition	-				
Updating	.200	-			
Flexibility	.344	.148	-		
Affective decision-making	.031	.077	-.007	-	
Delay of gratification	.034	.161	-.016	-.036	-

Note: I = inhibition, U = working-memory updating, F = cognitive flexibility, ADM = affective decision-making, D = delay of gratification.

The M*plus* output RESIDUAL was used to extract model estimated covariances and residuals for covariances. In a first step, model estimated covariances and residuals for covariances were added together. Then correlations of residuals were calculated using the following formula:

$$COR\ (I, U) = \frac{residual\ COV\ (I, U)}{\sqrt{Var(I) * Var\ (U)}}$$

No significance tests were available. However, the correlations table (S3) suggests that the three cool EF (I, U, F) typically showed higher residual correlations among one other than the two hot EF. Because of this, partial conditional dependence was assumed between the three cool EF in the next and final model step. By allowing the cool EF to correlate within our main analyses, the trend described here was confirmed by clearly significant associations.

Table S5

Bivariate Correlations of Profile Membership Probabilities (Gained from Latent Profile Analysis) and Self-Regulatory Outcomes

	1	2	3	4	5	6	7	8	9
1. *Low-inhibition* profile	-								
2. *Regulated-DM* profile	-.065**	-							
3. *Low-delay* profile	-.108***	-.151***	-						
4. *All-average* profile	-.340***	-.432***	-.699***	-					
5. Inhibitory control T2	-.101***	.000	-.030	.021	-				
6. Inhibitory control T3	-.064*	.012	-.022	.040	.611***	-			
7. Emotional reactivity T2	-.057*	.029	-.046	.047	.355***	.278***	-		
8. Emotional reactivity T3	-.023	.022	-.033	.024	.297***	.334***	.638***	-	
9. Planning / organizing T2	-.147***	-.008	-.021	.088**	.251***	.265***	.123***	.154***	-
10. Planning / organizing T3	-.084**	-.059*	.003	.074*	.215***	.275***	.190***	.211***	.617***

Note. Pairwise deletion of missing data, resulting in a range of n = 750 – 1,647, depending on variable modality (behavioral, parental or teacher report) and time point of collection (T1 – T3)

*p < .05, **p < .01, **p < .001

Figure S2

Mean Performance Across Five Executive Functions in Four Latent Profiles Assuming Partial Conditional Independence Across All Variables (N = 1,657), Model Entropy = .827

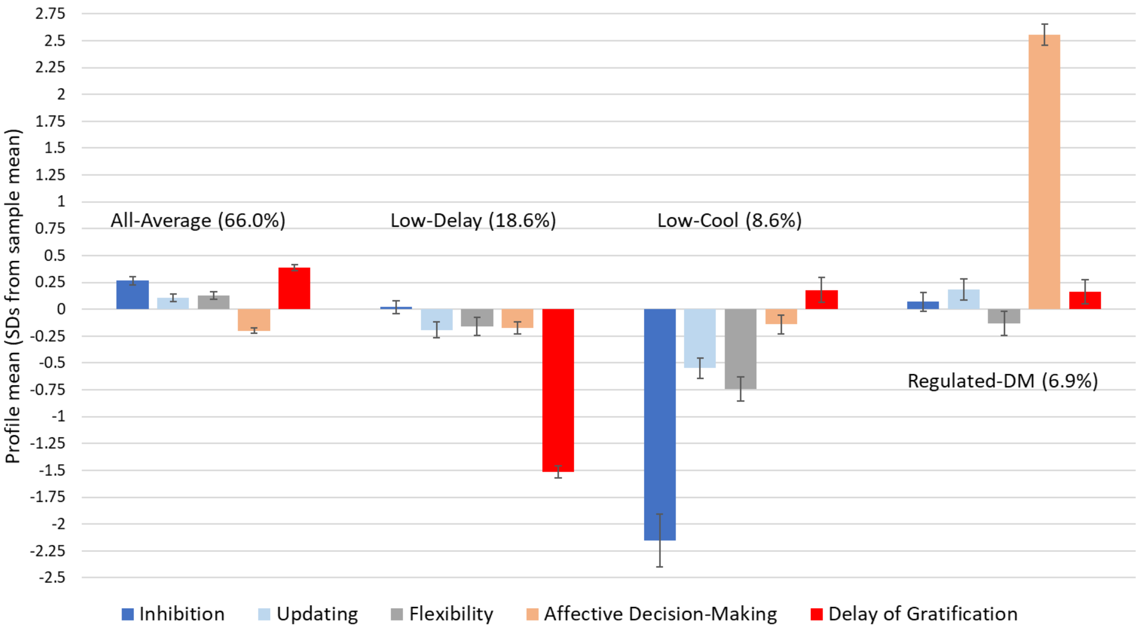

Table S5

Z-Standardized Means for Latent EF Profile Model Including Four Profiles and Assuming Partial Conditional Independence of All Variables

	All-Average (66.0%):	Low-Delay (18.6%):	Low-Inhibition (8.6%):	Regulated-DM (6.9%):
Inhibition (I)	0.267 (0.039)	0.020 (0.061)	-2.154 (0.247)	0.070 (0.088)
Updating (U)	0.106 (0.037)	-0.192 (0.074)	-0.548 (0.094)	0.184 (0.098)
Flexibility (F)	0.127 (0.037)	-0.157 (0.084)	-0.744 (0.113)	-0.131 (0.113)
Affective decision-making (ADM)	-0.199 (0.024)	-0.173 (0.054)	-0.142 (0.090)	2.556 (0.100)
Delay of gratification (D)	0.387 (0.029)	-1.512 (0.056)	0.180 (0.116)	0.161 (0.112)

The original 4-profile model with full restrictions on variance and covariance are presented here. Within this model, the *low-inhibition* profile showed an exaggerated pattern as compared to the profile accounting for correlations between residuals of the cool EF. Particularly, updating and flexibility showed reduced performance as compared to the final model with the best fit.

References

Grob, A., and Smolenski, C. (2005). *FEEL-KJ: Manual und Fragebogen zur Erhebung der Emotionsregulation bei Kindern und Jugendlichen [FEEL-KJ: Manual and questionnaire for the assessment of emotion regulation in children and adolescents]*. Bern: Huber

Rohlf, H. L., and Krahé, B. (2015). Assessing anger regulation in middle childhood: Development and validation of a behavioral observation measure. *Front. Psychol.* 6, 453. doi: 10.3389/fpsyg.2015.00453
